# Motor cortical plasticity induced by motor learning through mental practice

**DOI:** 10.3389/fnbeh.2015.00105

**Published:** 2015-04-28

**Authors:** Laura Avanzino, Nicolas Gueugneau, Ambra Bisio, Piero Ruggeri, Charalambos Papaxanthis, Marco Bove

**Affiliations:** ^1^Department of Experimental Medicine, Section of Human Physiology, University of GenoaGenoa, Italy; ^2^Université de Bourgogne, Unité de Formation et de Recherche en Sciences et Techniques des Activités Physiques et SportivesDijon, France; ^3^Laboratoire Institut National de la Santé et de la Recherche Médicale (INSERM), Unité 1093, Cognition, Action et Plasticité Sensorimotrice, Université de BourgogneDijon, France

**Keywords:** cortical plasticity, motor imagery, motor learning, long term potentiation, long term depression

## Abstract

Several investigations suggest that actual and mental actions trigger similar neural substrates. Motor learning via physical practice results in long-term potentiation (LTP)-like plasticity processes, namely potentiation of M1 and a temporary occlusion of additional LTP-like plasticity. However, whether this neuroplasticity process contributes to improve motor performance through mental practice remains to be determined. Here, we tested skill learning-dependent changes in primary motor cortex (M1) excitability and plasticity by means of transcranial magnetic stimulation (TMS) in subjects trained to physically execute or mentally perform a sequence of finger opposition movements. Before and after physical practice and motor-imagery practice, M1 excitability was evaluated by measuring the input-output (IO) curve of motor evoked potentials. M1 LTP and long-term depression (LTD)-like plasticity was assessed with paired-associative stimulation (PAS) of the median nerve and motor cortex using an interstimulus interval of 25 ms (PAS25) or 10 ms (PAS10), respectively. We found that even if after both practice sessions subjects significantly improved their movement speed, M1 excitability and plasticity were differentially influenced by the two practice sessions. First, we observed an increase in the slope of IO curve after physical but not after MI practice. Second, there was a reversal of the PAS25 effect from LTP-like plasticity to LTD-like plasticity following physical and MI practice. Third, LTD-like plasticity (PAS10 protocol) increased after physical practice, whilst it was occluded after MI practice. In conclusion, we demonstrated that MI practice lead to the development of neuroplasticity, as it affected the PAS25- and PAS10- induced plasticity in M1. These results, expanding the current knowledge on how MI training shapes M1 plasticity, might have a potential impact in rehabilitation.

## Introduction

Practice is fundamental for the acquisition of motor skills. Through repetition, movements are executed faster, accurately, and effortlessly (Willingham, 1998). Animal studies showed that motor learning leads to long-term potentiation (LTP) in the primary motor cortex (M1) (Sanes and Donoghue, 2000). This learning-induced LTP temporary occludes further potentiation, while enhancing long-term depression (LTD; Rioult-Pedotti et al., 1998, 2000, 2007; Monfils et al., 2004; Hodgson et al., 2005). Non-invasive techniques in humans also suggest that LTP-like plasticity is involved during motor learning. Paired-associative stimulation (PAS), consisting of transcranial magnetic stimulation (TMS) of the M1 combined with electrical stimulation of the median nerve, can be used to measure LTP-like and LTD-like effects (Stefan et al., 2000; Wolters et al., 2003). LTP-like effects are induced by an interstimulus interval of 25 ms (PAS25), while LTD-like effects by an interstimulus interval of 10 ms (PAS10). As in animals, a period of motor learning reversed or occluded LTP-like effects, whereas it either enhanced LTD-like effects or left them unchanged (Ziemann et al., 2004; Stefan et al., 2006; Rosenkranz et al., 2007).

Motor learning can occur by motor-imagery (MI) practice (Gentili et al., 2006, 2010; Lotze and Halsband, 2006; Avanzino et al., 2009); that is, by the internal simulation of a movement without any motor output. Evidence supports the hypothesis that internal forward models, which are neural processes that mimic the causal flow of the physical process by predicting the future sensorimotor state (e.g., position, velocity) given the efferent copy of the motor command and the current state, are involved in mental practice (Wolpert and Flanagan, 2001). Imagined and actual movements trigger similar motor representations (Jeannerod and Decety, 1995; Gentili et al., 2004; Gandrey et al., 2013) and share overlapping brain substrates (Grèzes and Decety, 2001; Jeannerod, 2001; Lotze and Halsband, 2006; Filimon et al., 2007; Hanakawa et al., 2008). Recent investigations have shown that motor imagery increases M1 excitability (Porro et al., 1996; Fadiga et al., 1998; Vargas et al., 2004; Gueugneau et al., 2013) and that motor cortex is functional relevant for motor learning by mental practice (Debarnot et al., 2011; Pelgrims et al., 2011; Foerster et al., 2013). Indeed, it has been demonstrated that M1 activity during motor imagery was positively correlated to improvements in accuracy in a precision grip task. The association between M1 activation during motor imagery with performance changes indicates that subjects with stronger M1 activation during motor imagery may benefit more from motor imagery training (Blefari et al., 2015).

In addition, repetitive mental practice can modulate neural circuits involved in the early stage of motor skill learning (Pascual-Leone et al., 1995). Recently, Zhang and coworkers assessed the resting-state functional connectivity before and after 2 weeks of motor imagery learning (Zhang et al., 2014). Cognitive and sensory resting state networks in multiple brain systems exhibited alterations at functional connectivity level after motor imagery learning.

Today, however, despite the promising effects of MI practice on skill learning and rehabilitation, the neural mechanisms underlying this effect are not completely elucidated. The present study was designed to explore changes in motor cortical plasticity and excitability induced by MI practice. In different days participants practiced a motor learning paradigm that consisted in executing or imagining a sequence of finger-tapping movements at maximal speed. Plasticity in M1 was induced by means of PAS protocols in two sets of experiments, testing long-term potentiation like (LTP) and long-term depression like (LTD) effects on cortical excitability. We found that MI practice leads to the development of neuroplasticity, i.e., it affects the subsequent induction of LTP/D-like plasticity in the human M1.

## Materials and Methods

### Subjects

Twenty-four, right-handed subjects (12 males, mean age: 23.5 ± 2.45 years) participated in this study. All were in good health, with normal or corrected vision and without any nervous, muscular or cognitive disorders. Right arm dominance was determined by means of the Edinburgh Handedness inventory (Oldfield, 1971). The Participants’ general motor imagery ability was evaluated by means of the Italian version of the Movement Imagery Questionnaire (MIQ-R) (Hall and Martin, 1997). The MIQ-R is an 8-item self-report questionnaire, in which participants rated the vividness of their mental representations using two 7-point scales (associated to visual and kinesthetic imagery): the score “7” means “really easy to feel/see”, whereas the score “1” corresponds to “really difficult to feel/see”. All participants showed good motor imagery abilities (mean ± SD: 47.1 ± 3.2; best score = 56, worst score = 8). The experimental protocol was approved by the ethics committee of the University of Genoa and was carried out in agreement with legal requirements and international norms (World Medical Association, 1964).

### Study Design

Participants were divided into two groups (12 males and 12 females per group) according to the Paired Associative Stimulation (PAS) protocol they would follow (GROUP_PAS25_ and GROUP_PAS10_) (for details see *PAS protocol section*). All took part to a first experimental session designed to test the effect of PAS25 and PAS10 on Motor Evoked Potentials (MEPs). Subjects were admitted to the subsequent experimental sessions if, in the first session, PAS10 had led to a decrease (GROUP_PAS10_) and PAS25 had led to an increase (GROUP_PAS25_) of MEP amplitudes. Of 24 subjects, 18 fulfilled this criterion and participated to the next experiments: 5 males and 4 females for GROUP_PAS25_ and 4 males and 5 females for GROUP_PAS10_ (see Table 1 for demographic characteristics).

**Table 1:** **Demographic and baseline TMS characteristics**.

	GROUP_PAS25_	GROUP_PAS10_
Gender (M/F)	5/4	4/5
Age ± S.D.	23.3 ± 2.4	23.7 ± 2.6
First session RMT ± SD	39.5 ± 4.8	42.3 ± 5.7
First session S1mV ± SD	44.4 ± 4.9	46.1 ± 6.7

*RMT, resting motor threshold; S1mV, stimulus intensity needed to evoke a MEP of approximately 0.8−1 mV peak-to-peak amplitude; SD, standard deviation*.

In the other two sessions, separated by at least 1 week, the effect of PAS25 or PAS10 on cortical excitability was evaluated after a physical practice session or a MI practice session. The order of the two sessions was counterbalanced between the participants.

**Figure 1:**
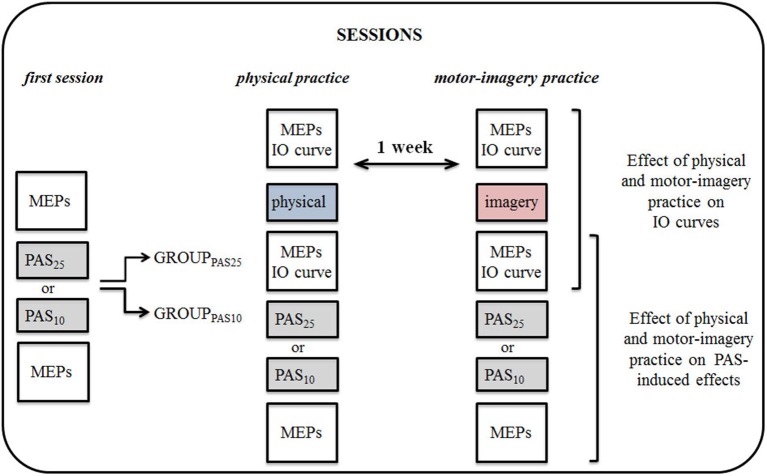
**Experimental protocol**. By means of transcranial magnetic stimulation (TMS), we tested the effect of physical practice (physical) and motor imagery practice (imagery) on corticospinal excitability and on Paired associative stimulation (PAS) protocol induced effects. Participants were divided into two groups, each participating in a set of experiments, testing the effect of PAS25 and PAS10 separately. All experiments and sessions of physical practice or motor-imagery (MI) practice were performed in the morning. MEPs, motor evoked potentials; IO, input-output curve.

MEPs and Input-Output (IO) curve (for details see *TMS parameters* section) were measured before and after the physical practice or MI practice sessions. Immediately after the practice sessions, the PAS protocol and the subsequent measurement of MEPs were applied. The experimental protocol is shown in Figure 1.

### Physical Practice and Motor-Imagery Practice Sessions

Participants were seated in a comfortable chair in a quiet room and wore a sensor-engineered glove (Glove Analyzer System (GAS), ETT S.p.A., Italy) on their right hand. In the physical practice session, they were instructed to execute as fast as possible the following motor sequence of finger movements: opposition of thumb to index, medium, ring and little fingers. An eyes-closed paradigm was chosen to avoid possible confounding effects due to the integration of acoustic and visual information. All the participants had a short familiarization session during which they had to realize few trials of the task at a natural velocity. After 3–5 finger sequences, all participants reported being comfortable with the task. Then, physical practice consisted in performing as fast as possible 15 blocks of 5 sequences with 10 s rest between the blocks (total number of finger movements: 300 (4 fingers × 5 sequences × 15 blocks). The MI practice consisted in imagining the same task. Precisely, participants were asked to imagine themselves performing the movement (“kinesthetic imagery” or first-person perspective), as they would actually do (Gentili et al., 2010). They were asked to feel the motion of their fingers and the contact between the distal phalanx of the thumb and those of the other fingers. Imagining a movement in the first person is a necessary condition to engage the motor system (Stinear et al., 2006). After a short pre-session briefing, and few trials of mentally simulated movements at a natural velocity, all participants declared being able to generate mental movements without difficulties. Participants were continuously encouraged by the experimenter to increase their movement speed throughout the training sessions.

To evaluate the effect of physical practice and MI practice on motor performance, participants performed before the training session (pre-test) and after it (post-test), one block of 5 sequences at their maximum speed. Data from glove were processed with a customized software and the finger movement performance rate was extracted and expressed in Hz.

All subjects became faster during the training sessions (either physical practice and MI practice). We quantified the learning effect by measuring the increase in the value of finger movement performance rate in the post-test block of 5 finger sequences. To verify that MI training was really proficient to induce motor learning, 8 additional right-handed subjects (4 males and 4 females, mean age 22.6 ± 4.5 years) were involved in a control experiment. It consisted of performing two blocks of 5 finger sequences (separated by a rest period of about 10 min) at their maximum speed, with the aim of comparing these results with those obtained with the MI protocol.

### TMS Parameters

Single-pulses were delivered using a Magstim 200 stimulator (Magstim Co., Whitland, Wales, UK) with a monophasic current waveform connected to a figure-of-eight-shaped coil (external diameter of each loop, 9 cm) held tangentially to the scalp. The center of the junction of the coil was placed over the hand area of the left primary motor cortex (M1) at the optimal position (hot spot) to elicit MEPs in the contralateral abductor pollicis brevis (APB), with the handle pointing backwards and ~45° away from the midline. With this coil orientation, the induced current flowed in an anterior–medial direction approximately perpendicular to the central sulcus. The optimal coil location was searched by slightly moving the coil over the left M1 area until MEPs of maximal amplitude and lowest threshold in the right APB were elicited. The exact coil position was marked by an inking pen to ensure an accurate positioning of the coil throughout the experiment. At the beginning of each experiment, the stimulus intensity needed to evoke MEPs of approximately 0.8−1 mV peak-to-peak amplitude was defined (S1mV). This intensity was used to evaluate MEPs changes before and after PAS protocol, as the reference to record the IO curve, and to make the PAS protocol (see below). During the IO curve the intensities of single TMS stimuli were expressed as a percentage of S1mV intensity. Twelve MEPs were recorded with 70%, 80%, 90%, 100% (S1mV), 110%, 120%, 130% and 140% stimulus intensities. For each participant, the peak-to-peak MEP amplitude on single trials was used to calculate the mean MEP amplitude at each stimulus intensity.

### EMG Recording

EMG was recorded through surface electrodes from the right APB muscle using pairs of Ag-AgCl electrodes. The EMG signals were amplified (×1000), filtered with a bandwidth ranging from 10 Hz to 1 kHz, analog-to-digital converted at a sampling frequency of 2 kHz and fed into a personal computer by means of the MP100 acquisition system (BIOPAC Systems Inc., Santa Barbara, CA, USA). Each recording epoch lasted 400 ms, of which 100 ms preceded the TMS. We cautiously controlled the EMG activity in real-time during MI training to ensure that the mentally simulated movements were not contaminated by muscle activity. Muscle activity in all mental trials was lower than 10 μV and not different from muscle activity at rest (*p* > 0.1).

### PAS Protocol

The PAS protocol consisted of electrical stimuli of the right median nerve at the wrist level paired with single TMS pulses over the hotspot of the APB muscle area of the left hemisphere at a rate of 0.25 Hz (Ziemann et al., 2004). The electrical stimulation was applied through a bipolar electrode (cathode proximal) using a square-wave pulse (duration, 0.2 ms) at an intensity of three times the perceptual threshold (Digitimer D180 high voltage electric stimulator). The TMS was delivered in the way described above at S1mV stimulus intensity. Two hundred paired stimulations were applied. For PAS25 the inter-stimulus interval between peripheral and TMS stimuli was of 25 ms, whereas for PAS10 it was of 10 ms. The former has been previously shown to induce a long-lasting MEP increase (Stefan et al., 2000, 2002) and the latter a MEP decrease (Wolters et al., 2003). Participants were instructed to look at their stimulated hand and count the peripheral electrical stimuli they perceived so as to standardize the visual attentional load during the PAS protocols (Kamke et al., 2012). The MEPs evoked in the APB were displayed online during the intervention to control for the correct coil position and stored for off-line analysis.

### Statistical Analysis

We checked that variables were normally distributed (*Shapiro-Wilk W test*) and that sphericity was respected (Mauchly tests).

To evaluate motor performance improvements (i.e., increase in movement rate) induced by practice, we run a three-way RM-ANOVA (2 × 2 × 2) with PRACTICE (physical and MI) and TIME (pre-test and post-test) as within-subject factors, and GROUP (GROUP_PAS25_ and GROUP_PAS10_) as between-subjects factor. Further, Pearson’s correlation was applied to assess any correlation between individual changes in movement rate induced by MI practice and individual scores at the MIQ-R questionnaire. Changes in movement rate were evaluated as follows: (post-test − pre-test)/post-test × 100).

Increase of movement speed gained after MI training in GROUP_PAS25_ and GROUP_PAS10_ was finally compared with that obtained in the control group, which performed two blocks of 5 sequences at their maximum speed with a rest period of 10 min between the blocks, by means of a one-way ANOVA with the factor GROUP (GROUP_PAS25_, GROUP_PAS10_ and control experiment GROUP) as main factor.

To evaluate the effect of physical practice and MI practice on IO curves, we calculated the slopes of the IO curves, defined as the steepness of the linear regression line through the given data points between 90% and 130% S1mV. IO slope values were entered in a three-way RM-ANOVA (2 × 2 × 2) with PRACTICE and TIME as within-subjects factor and GROUP as between-subjects factor.

The influence of physical practice and MI practice on PAS-induced effects was assessed by entering MEPs values corresponding to the S1mV in a two-way RM-ANOVA (3 × 2) with SESSION (first session, physical practice, MI practice) and TIME (before PAS and after PAS) as within-subjects factors. Because the PAS effect is described relative to the measures taken before, the choice of baseline MEP, either before physical-practice or MI-practice, would influence the statistical results. To control for this we took a conservative approach: RM-ANOVAs that included the factor TIME were calculated in two versions. One used the MEPs recorded after practice (i.e., immediately before PAS) as baseline and the second used MEPs before practice as baseline. Only those results are reported that turned out to be significant in both versions of the statistics (Rosenkranz et al., 2007). All the above statistical analyses were performed separately for the PAS25 and the PAS10 groups. *Post hoc* analysis of significant interactions was performed by means of *t*-tests applying the Bonferroni correction for multiple comparisons when necessary. *p*-values of 0.05 were considered as threshold for statistical significance. Statistical analysis was performed with SPSS 13.0.

## Results

### Motor Performance

**Figure 2:**
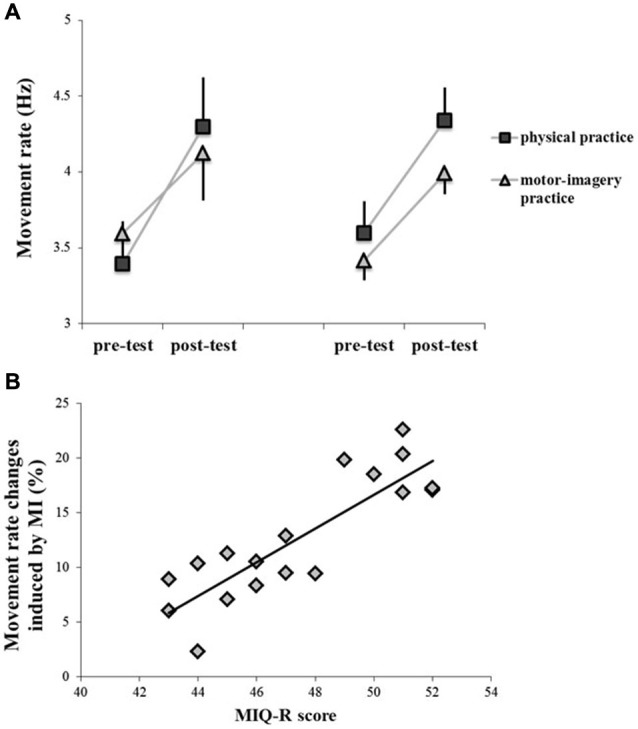
**Behavioral data, showing the rate of execution of sequential finger tapping movements before (pre-test) and after (post-test) the different types of training (physical practice and motor-imagery practice) (panel A)**. Data of both GROUP_PAS10_ (left side) and GROUP_PAS25_ (right side) are shown. Vertical bars indicate SE. Panel **(B)** shows the correlation between individual changes in movement rate induced by motor-imagery (MI) practice and individual scores at the MIQ-R questionnaire. Changes in movement rate (*Y*-axis) were evaluated as follows:(post-test − pre-test)/post-test × 100) (*r* = 0.85; *p* < 0.001).

Participants physically and mentally performed 300 sequential finger movements at maximum speed in two separate days. We found that the rate of execution of sequential finger-tapping movements significantly increased after both practice sessions (main effect of TIME, *F*_1,16_ = 339.47; *p* < 0.001). We found neither a main effect of GROUP (*F*_1,16_ = 0.043; *p* = 0.83), nor an interaction GROUP * PRACTICE (*F*_1,16_ = 1.79; *p* = 0.19) or GROUP * PRACTICE * TIME (*F*_1,16_ = 0.010; *p* = 0.19). However, there was a PRACTICE by TIME interaction effect (*F*_1,16_ = 20.55; *p* < 0.001). *Post hoc* analysis revealed that although both practice sessions were able to improve motor performance (pre-test vs. post-test; *p* < 0.001 for both comparisons), the increase of movement rate in post-test was higher after physical practice than after MI practice (*p* = 0.038). Note that pre-test values were comparable between physical practice and MI practice (*p* = 0.93) (Figure 2A).

Correlation analysis between individual changes in movement rate induced by MI practice and individual scores at the MIQ-R questionnaire showed that the more the subjects were good imagery performers the more MI practice was effective in modifying motor performance (*r* = 0.85; *p* < 0.001) (Figure 2B).

Finally, one-way ANOVA showed a main effect of GROUP (*F*_2,11_ = 4.11; *p* = 0.042) when comparing changes in movement rate induced by MI and physical practice with those measured in the control experiment, in which participants did not perform any type of training. *Post hoc* analysis showed that the increase in movement rate was significantly higher in the MI condition (GROUP_PAS25_, 14.61 ± 6.2%; GROUP_PAS10_, 13.54 ± 5.3%) than in the control experiment (control GROUP, 6.82 ± 3.5%) (GROUP_PAS25_ vs. control, *p* = 0.028; GROUP_PAS10_ vs. control, *p* = 0.040).

### Effect of Physical Practice and Motor-Imagery Practice on IO Curves

**Figure 3:**
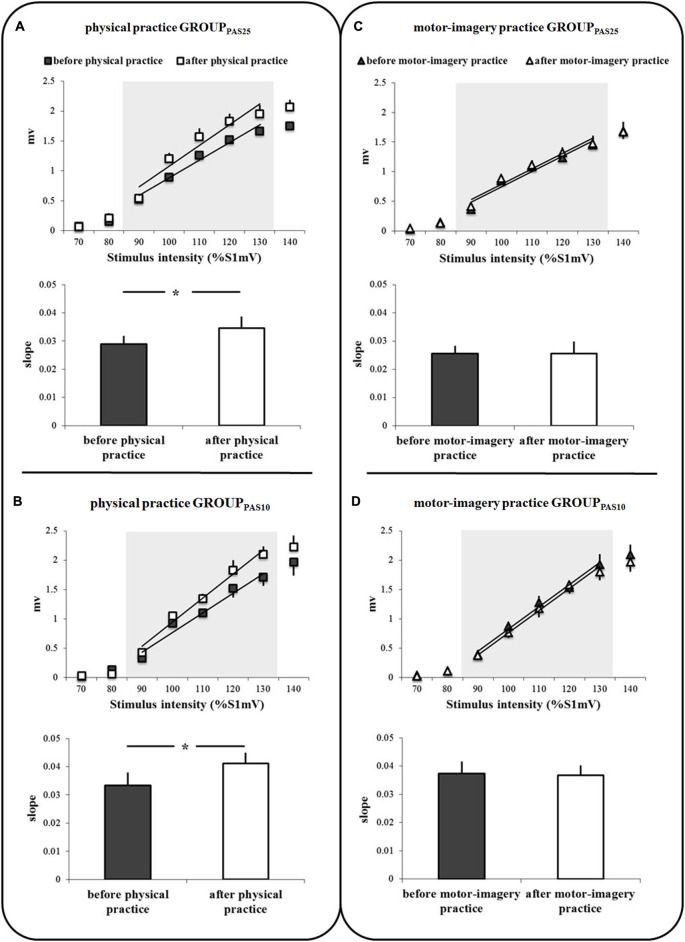
**IO curves measured in the abductor pollicis brevis (APB) muscle, before and after physical practice (A,B) and before and after motor-imagery practice (C,D)**. Data of both GROUP_PAS25_ **(A,C)** and GROUP_PAS10_ **(B,D)** are shown. Trend lines indicate the linear fit applied to the range from 90% to 130% S1mV above RMT as indicated by the shaded area. The slopes of the IO curves are depicted in the inferior part of each panel **(A,D)**. The slope was estimated from the linear part of the IO curve between 90% and 130% S1mV above RMT. Vertical bars indicate SE. Asterisks indicate the level of significance (* *p* < 0.05).

Figures 3A–D shows the IO curves obtained in the APB before and after the physical practice and MI practice sessions in the two groups of participants (GROUP_PAS25_ and GROUP_PAS10_). A clear difference in IO curves appeared for physical practice only. ANOVA on the slope data showed a significant PRACTICE by TIME interaction (*F*_1,16_ = 13.85; *p* = 0.002). *Post hoc* analysis revealed that before both practice sessions, the slopes of IO curves were similar (*p* = 0.89). IO curves were significantly steeper after physical practice (*p* = 0.001), but not after MI practice (*p* = 0.86). We found neither a main effect of GROUP (*F*_1,16_ = 3.11; *p* = 0.097), nor an interaction GROUP * TIME (*F*_1,16_ = 0.08; *p* = 0.77) or GROUP * PRACTICE * TIME (*F*_1,16_ = 0.54; *p* = 0.47).

### Effect of Physical Practice and Motor-Imagery Practice on PAS-Induced Effects

**Figure 4:**
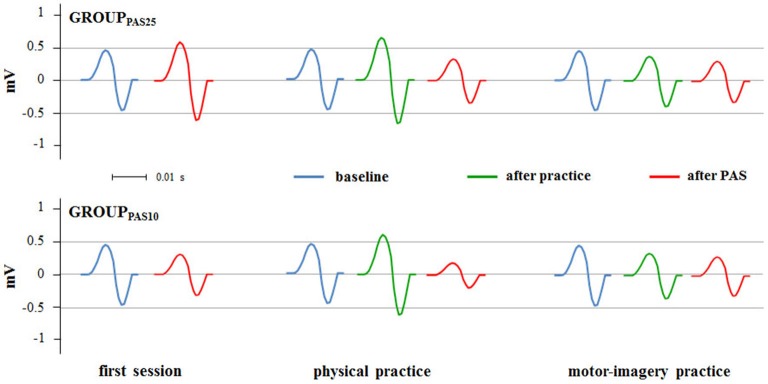
**PAS effects on MEPs amplitude in different experimental sessions (first session, physical practice and motor-imagery practice)**. Raw MEPs obtained from two single representative subjects belonging the GROUP_PAS25_ and GROUP_PAS10_ are shown.

**Figure 5:**
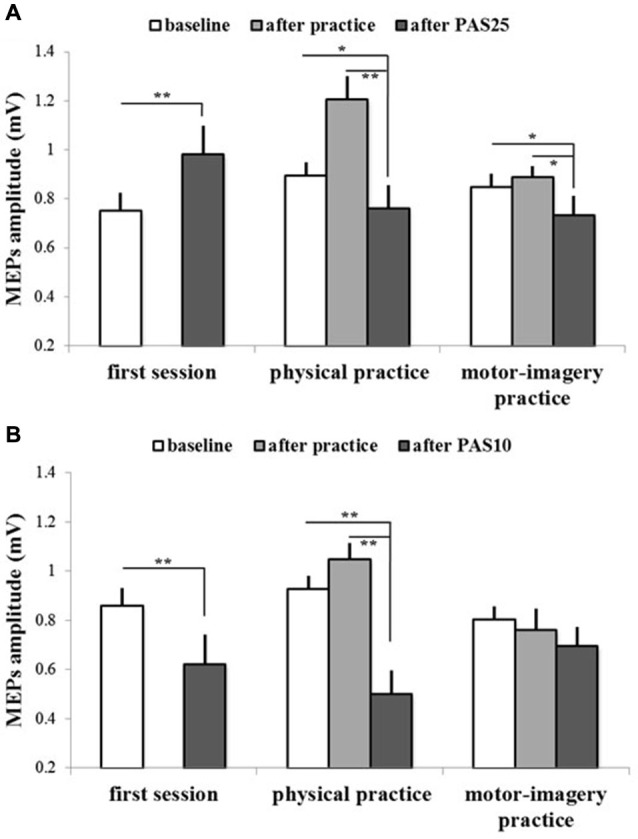
**PAS effects on mean MEPs amplitude in different experimental sessions (first session, physical practice and motor-imagery practice) for both GROUP_PAS25_ (A) and GROUP_PAS10_ (B)**. Data obtained before and after practice and after PAS protocol administration are shown. Vertical bars indicate SE. Asterisks indicate significant difference between MEPs before PAS and MEPs after PAS when interaction of SESSION * TIME was statistically significant (*p < 0.05; **p < 0.01).

Figures 4, 5 show the specific impact of physical practice and motor-imagery practice on PAS-induced effects. Figure 5A shows PAS effects on MEPs amplitude for the GROUP_PAS25_. ANOVA showed a significant interaction SESSION * TIME (*F*_2,16_ > 7.75; *p* < 0.004). *Post hoc* analysis revealed that on the first session the administration of PAS25 protocol increased the MEPs amplitude (before PAS25 vs. after PAS25, *p* = 0.006). On the contrary, after both physical practice and MI practice, the PAS25 protocol significantly reduced MEPs amplitude (for physical practice: *p* < 0.040; for MI practice: *p* < 0.045).

Figure 5B shows PAS effects on MEPs amplitude for the GROUP_PAS10_. ANOVA showed a significant interaction SESSION * TIME (*F*_2,16_ > 14.38; *p* < 0.001). *Post hoc* analysis revealed that the administration of PAS10 protocol decreased the MEPs amplitude both on the first session (before PAS10 vs. after PAS10, *p* = 0.004) and after physical practice (*p* < 0.001), whereas no effect was observed after MI practice (*p* > 0.095). Further, a stronger reduction of MEPs amplitude was observed when PAS10 was administered after physical practice with respect to that induced by PAS10 in the first session (*p* = 0.048).

## Discussion

In the current study, we investigated M1 plasticity induced by physical or MI practice. We found that after both practice sessions, subjects significantly improved their movement speed. However, we observed an increase in the slope of IO curve after physical practice, but not after MI practice. Further, there was a reversal of the PAS25 effect from LTP-like plasticity to LTD-like plasticity following physical and MI practice. LTD-like plasticity (PAS10 protocol) significantly increased after physical practice, whilst it was occluded after MI practice.

### Motor Learning by Physical Practice and Motor-Imagery Practice

In accordance with previous studies (Gentili et al., 2006, 2010; Avanzino et al., 2009), we found that both physical practice and, even if to a lesser extent, MI practice, significantly increased movement speed. Further, individual motor imagery ability positively correlated with changes in movement rate induced by MI practice. In other words, the better performers at the MIQ-R were those who increased more the finger movement rate after MI practice.

The different amount of learning between physical practice and MI practice, already observed in previous studies (Gentili et al., 2006, 2010), might be explained by sensorimotor mechanisms that operated differently during the two training methods. Motor learning by physical-practice involves both motor and sensory processes (Haith and Krakauer, 2013). Indeed, during physical practice we face the dual challenge of determining and refining the somatosensory goals of our movements and establishing the best motor commands to achieve our ends (Vahdat et al., 2014). The two processes typically proceed in parallel, and skill acquisition is a reflection of changes in sensory systems and in the brain motor areas. On the other hand, mental simulation during MI practice is generated by internal forward models, which predict the future state given the actual state and the efference copy of the motor command (Wolpert et al., 1998). Because state estimation derives from the forward model alone, the training signal is presumably less accurate and less precise than during physical training, thus leading to a less amount of learning. It’s worthy to note that it has already been reported that the extent of the vividness of a motor image is associated with the pattern and/or level of neural activation in motor and related areas (Williams et al., 2012). Thus, in accordance with our behavioral findings, differences in learning effect between the two types of training may also been explained by individual differences in motor imagery ability.

### Effect of Physical Practice and Motor-Imagery Practice on IO Curve

From a neurophysiological point of view, only physical practice significantly increased the steepness of the IO curve. This training-dependent increase in cortico-motoneuronal excitability is in line with previous studies (Devanne et al., 1997; Lotze et al., 2003). It may indicate that neurons with higher threshold to TMS (including neurons located at a longer distance from the center of the coil) exhibit large excitability changes after physical training, i.e., that there is an expansion of the representation area of the APB muscle in M1 (Ridding and Rothwell, 1999).

Conversely, MI practice did not change the slope of the IO curve. This finding could be explained by the weaker activation of the excitatory circuits within M1 during motor imagery. It has been consistently shown with different neurophysiological and neuroimaging techniques that M1 activation is significantly smaller during imagined compared with actual movements (Porro et al., 1996; Schnitzler et al., 1997; Fadiga et al., 1998; Gueugneau et al., 2013). An explanation of this phenomenon could be the increase or absence of release of the inhibitory drive over M1 originating from many other cortical areas that prevents motor execution during mental practice (Pelgrims et al., 2011; Guillot et al., 2012). Indeed, interactions between the pre-motor areas, the posterior parietal lobe, and M1 are facilitatory during actual movements and inhibitory during imagined movements (Solodkin et al., 2004; Kasess et al., 2008).

### Effect of Physical Practice and Motor-Imagery Practice on PAS Induced Effects

Physical practice influenced the PAS25 effect by reversing the LTP-like plasticity towards a LTD-like plasticity and influenced the PAS10 effect by enhancing the LTD-like plasticity. Our findings confirm those of previous studies (Ziemann et al., 2004; Stefan et al., 2006; Rosenkranz et al., 2007), in which it has been suggested that physical activity reduces the probability for LTP-induction and increases the probability for LTD-induction. In our study, the novel finding is that MI practice also lead to the development of neuroplasticity, as it affected the PAS25- and PAS10- induced plasticity in M1. This finding further points out the role of motor-related areas, and especially that of M1, in motor learning by mental practice (Debarnot et al., 2011; Pelgrims et al., 2011).

Motor learning by means of MI practice affected the PAS25- and PAS10- induced plasticity in a different way compared with physical practice. In particular, whereas the MI training effect on PAS25 protocol was in the same direction as that of physical practice; i.e., reversing the LTP-like effect towards a LTD-like plasticity, when PAS10 was applied after MI training, we observed an occlusion of LTD-like plasticity. Specifically, after MI training, PAS10 protocol was unable to induce any change in MEPs amplitude, neither inhibition nor excitation.

For LTP and LTD induction, the critical variable in determining the sign of the synaptic modification appears to be the level of postsynaptic depolarization and the resulting pattern of postsynaptic increases in Ca^2+^ levels. A transient inflow of high amounts of Ca^2+^ into the neuron triggers LTP, while LTD is induced by a modest but more sustained elevation in postsynaptic Ca^2+^ levels (Kirkwood and Bear, 1995; Bi and Poo, 2001). However, a moderate postsynaptic Ca^2+^ elevation that lasts too short for inducing LTD and is too small for inducing LTP-like plasticity can result in a plasticity occlusion (Quartarone et al., 2006).

Theoretical concepts and experimental works on synaptic modification (Bienenstock et al., 1982; Kirkwood and Bear, 1995; Toyoizumi et al., 2005) suggest that the threshold for inducing either LTP or LTD at a synapse depends on the history of synaptic activity. Following the activity-dependent plasticity model, high synaptic activity in M1, as it can occur after physical practice training, makes LTP harder to be induced and LTD easier to be induced (Bienenstock et al., 1982). We might speculate that motor imagery practice induced a different modulation of the M1 synaptic activity than physical practice. Indeed, during physical practice, the sensorimotor pathway is strongly engaged and inhibitory mechanisms are partially involved (Kang et al., 2012; Shin et al., 2012; Vahdat et al., 2014). Differently, during motor imagery practice, sensory feedback is not primarily involved and cortico-cortical inhibition has a functional role in order to prevent overt movements. Our hypothesis is that the influence exerted by MI practice on PAS25 and PAS10 plasticity protocol effects in M1 might depend on the different effect exerted by the two practice protocols on M1 neural circuits (as demonstrated by the IO curve results). Indeed, we already discussed that only physical practice, and not motor imagery practice, significantly increased the steepness of the IO curve. This could be possibly due to the involvement of inhibitory circuits in M1, to the absence of movement-related sensory feedback, and to the lesser activation of M1 during MI practice than physical practice (Porro et al., 1996). This may suggest that when we applied the PAS25 and PAS10 plasticity protocol in the physical practice and motor imagery practice sessions, neuronal activity in M1 was differently modulated according to the type of previous training, thus influencing the LTP and LTD-like plasticity induction in the motor cortical representation of the right APB muscle. However, we are aware that these considerations are still theoretical and future studies should address the role of GABAergic intracortical inhibition in motor learning via motor imagery in order to strengthen our hypothesis.

In synopsis, our findings show that motor learning by means of MI practice lead to the development of neuroplasticity in the human primary motor cortex. Further, these results, expanding the current knowledge on how MI training shapes cortical plasticity, have a potential impact when this technique is applied in a rehabilitative setting.

It is worth to note that a recent study showed that, when tested with TMS, cortico-spinal excitability similarly increased during voluntary muscular contraction and during motor imagery combined with functional electrical stimulation and that this increase was larger than that observed during motor imagery alone (Kaneko et al., 2014). This finding highlights that when combined sensory input to motor imagery may be more effective in modulating M1 excitability than when administered alone. A possible development of our study might be to investigate M1 plasticity induced by motor imagery combined interventions that theoretically may be as effective as physical practice.

## Conflict of Interest Statement

The authors declare that the research was conducted in the absence of any commercial or financial relationships that could be construed as a potential conflict of interest.

## Abbreviations

LTP: Long-term potentiation
LTD: Long-term depression
M1: primary motor cortex
IO: input-output
PAS: paired associative stimulation
TMS: transcranial magnetic stimulation
MEP: motor evoked potential
APB: abductor pollicis brevis.

## References

[B1] Avanzino L. Giannini A. Tacchino A. Pelosin E. Ruggeri P. Bove M. (2009). Motor imagery influences the execution of repetitive finger opposition movements. Neurosci. Lett. 466, 11–15. 10.1016/j.neulet.2009.09.036 19770024

[B2] Bi G. Poo M. (2001). Synaptic modification by correlated activity: Hebb’s postulate revisited. Annu. Rev. Neurosci. 24, 139–166. 10.1146/annurev.neuro.24.1.139 11283308

[B3] Bienenstock E. L. Cooper L. N. Munro P. W. (1982). Theory for the development of neuron selectivity: orientation specificity and binocular interaction in visual cortex. J. Neurosci. 2, 32–48. 7054394 10.1523/JNEUROSCI.02-01-00032.1982 PMC6564292

[B4] Blefari M. L. Sulzer J. Hepp-Reymond M.-C. Kollias S. Gassert R. (2015). Improvement in precision grip force control with self-modulation of primary motor cortex during motor imagery. Front. Behav. Neurosci. 9:18. 10.3389/fnbeh.2015.00018 25762907 PMC4327737

[B5] Debarnot U. Clerget E. Olivier E. (2011). Role of the primary motor cortex in the early boost in performance following mental imagery training. PLoS One 6:e26717. 10.1371/journal.pone.0026717 22046337 PMC3202558

[B6] Devanne H. Lavoie B. A. Capaday C. (1997). Input-output properties and gain changes in the human corticospinal pathway. Exp. Brain Res. 114, 329–338. 10.1007/pl00005641 9166922

[B7] Fadiga L. Buccino G. Craighero L. Fogassi L. Gallese V. Pavesi G. (1998). Corticospinal excitability is specifically modulated by motor imagery: a magnetic stimulation study. Neuropsychologia 37, 147–158. 10.1016/s0028-3932(98)00089-x 10080372

[B8] Filimon F. Nelson J. D. Hagler D. J. Sereno M. I. (2007). Human cortical representations for reaching: mirror neurons for execution, observation and imagery. Neuroimage 37, 1315–1328. 10.1016/j.neuroimage.2007.06.008 17689268 PMC2045689

[B9] Foerster A. Rocha S. Wiesiolek C. Chagas A. P. Machado G. Silva E.. (2013). Site-specific effects of mental practice combined with transcranial direct current stimulation on motor learning. Eur. J. Neurosci. 37, 786–794. 10.1111/ejn.12079 23279569

[B10] Gandrey P. Paizis C. Karathanasis V. Gueugneau N. Papaxanthis C. (2013). Dominant vs. nondominant arm advantage in mentally simulated actions in right handers. J. Neurophysiol. 110, 2887–2894. 10.1152/jn.00123.2013 24089396

[B11] Gentili R. Cahouet V. Ballay Y. Papaxanthis C. (2004). Inertial properties of the arm are accurately predicted during motor imagery. Behav. Brain Res. 155, 231–239. 10.1016/j.bbr.2004.04.027 15364482

[B12] Gentili R. Han C. E. Schweighofer N. Papaxanthis C. (2010). Motor learning without doing: trial-by-trial improvement in motor performance during mental training. J. Neurophysiol. 104, 774–783. 10.1152/jn.00257.2010 20538766

[B13] Gentili R. Papaxanthis C. Pozzo T. (2006). Improvement and generalization of arm motor performance through motor imagery practice. Neuroscience 137, 761–772. 10.1016/j.neuroscience.2005.10.013 16338093

[B14] Grèzes J. Decety J. (2001). Functional anatomy of execution, mental simulation, observation and verb generation of actions: a meta-analysis. Hum. Brain Mapp. 12, 1–19. 10.1002/1097-0193(200101)12:1<1::aid-hbm10>3.0.co;2-v 11198101 PMC6872039

[B15] Gueugneau N. Bove M. Avanzino L. Jacquin A. Pozzo T. Papaxanthis C. (2013). Interhemispheric inhibition during mental actions of different complexity. PLoS One 8:e56973. 10.1371/journal.pone.0056973 23451125 PMC3581568

[B16] Guillot A. Di Rienzo F. MacIntyre T. Moran A. Collet C. (2012). Imagining is not doing but involves specific motor commands: a review of experimental data related to motor inhibition. Front. Hum. Neurosci. 6:247. 10.3389/fnhum.2012.00247 22973214 PMC3433680

[B17] Haith A. M. Krakauer J. W. (2013). Model-based and model-free mechanisms of human motor learning. Adv. Exp. Med. Biol. 782, 1–21. 10.1007/978-1-4614-5465-6_1 23296478 PMC3570165

[B18] Hall C. R. Martin K. A. (1997). Measuring movement imagery abilities: a revision of the movement imagery questionnaire. J. Ment. Imag. 21, 143–154.

[B19] Hanakawa T. Dimyan M. A. Hallett M. (2008). Motor planning, imagery and execution in the distributed motor network: a time-course study with functional MRI. Cereb. Cortex 18, 2775–2788. 10.1093/cercor/bhn036 18359777 PMC2583155

[B20] Hodgson R. A. Ji Z. Standish S. Boyd-Hodgson T. E. Henderson A. K. Racine R. J. (2005). Training-induced and electrically induced potentiation in the neocortex. Neurobiol. Learn. Mem. 83, 22–32. 10.1016/j.nlm.2004.07.001 15607685

[B21] Jeannerod M. (2001). Neural simulation of action: a unifying mechanism for motor cognition. Neuroimage 14, S103–S109. 10.1006/nimg.2001.0832 11373140

[B22] Jeannerod M. Decety J. (1995). Mental motor imagery: a window into the representational stages of action. Curr. Opin. Neurobiol. 5, 727–732. 10.1016/0959-4388(95)80099-9 8805419

[B23] Kamke M. R. Hall M. G. Lye H. F. Sale M. V. Fenlon L. R. Carroll T. J.. (2012). Visual attentional load influences plasticity in the human motor cortex. J. Neurosci. 32, 7001–7008. 10.1523/JNEUROSCI.1028-12.2012 22593068 PMC6622206

[B24] Kaneko F. Hayami T. Aoyama T. Kizuka T. (2014). Motor imagery and electrical stimulation reproduce corticospinal excitability at levels similar to voluntary muscle contraction. J. Neuroeng. Rehabil. 11:94. 10.1186/1743-0003-11-94 24902891 PMC4113028

[B25] Kang S. Y. Hallett M. Sohn Y. H. (2012). Synchronized finger exercise reduces surround inhibition. Clin. Neurophysiol. 123, 2227–2231. 10.1016/j.clinph.2012.04.019 22608486

[B26] Kasess C. H. Windischberger C. Cunnington R. Lanzenberger R. Pezawas L. Moser E. (2008). The suppressive influence of SMA on M1 in motor imagery revealed by fMRI and dynamic causal modeling. Neuroimage 40, 828–837. 10.1016/j.neuroimage.2007.11.040 18234512

[B27] Kirkwood A. Bear M. F. (1995). Elementary forms of synaptic plasticity in the visual cortex. Biol. Res. 28, 73–80. 8728822

[B28] Lotze M. Braun C. Birbaumer N. Anders S. Cohen L. G. (2003). Motor learning elicited by voluntary drive. Brain 126, 866–872. 10.1093/brain/awg079 12615644

[B29] Lotze M. Halsband U. (2006). Motor imagery. J. Physiol. Paris 99, 386–395. 10.1016/j.jphysparis.2006.03.012 16716573

[B30] Monfils M. H. VandenBerg P. M. Kleim J. A. Teskey G. C. (2004). Long-term potentiation induces expanded movement representations and dendritic hypertrophy in layer V of rat sensorimotor neocortex. Cereb. Cortex 14, 586–593. 10.1093/cercor/bhh020 15054074

[B31] Oldfield R. C. (1971). The assessment and analysis of handedness: the Edinburgh inventory. Neuropsychologia 9, 97–113. 10.1016/0028-3932(71)90067-4 5146491

[B32] Pascual-Leone A. Grafman J. Hallett M. (1995). Procedural learning and prefrontal cortex. Ann. N Y Acad. Sci. 769, 61–70. 10.1111/j.1749-6632.1995.tb38131.x 8595044

[B33] Pelgrims B. Michaux N. Olivier E. Andres M. (2011). Contribution of the primary motor cortex to motor imagery: a subthreshold TMS study. Hum. Brain Mapp. 32, 1471–1482. 10.1002/hbm.21121 21077146 PMC6870368

[B34] Porro C. A. Francescato M. P. Cettolo V. Diamond M. E. Baraldi P. Zuiani C.. (1996). Primary motor and sensory cortex activation during motor performance and motor imagery: a functional magnetic resonance imaging study. J. Neurosci. 16, 7688–7698. 8922425 10.1523/JNEUROSCI.16-23-07688.1996 PMC6579073

[B35] Quartarone A. Rizzo V. Bagnato S. Morgante F. Sant’Angelo A. Girlanda P.. (2006). Rapid-rate paired associative stimulation of the median nerve and motor cortex can produce long-lasting changes in motor cortical excitability in humans. J. Physiol. 575, 657–670. 10.1113/jphysiol.2006.114025 16825301 PMC1819453

[B36] Ridding M. C. Rothwell J. C. (1999). Afferent input and cortical organisation: a study with magnetic stimulation. Exp. Brain Res. 126, 536–544. 10.1007/s002210050762 10422717

[B37] Rioult-Pedotti M.-S. Donoghue J. P. Dunaevsky A. (2007). Plasticity of the synaptic modification range. J. Neurophysiol. 98, 3688–3695. 10.1152/jn.00164.2007 17913995

[B38] Rioult-Pedotti M. S. Friedman D. Donoghue J. P. (2000). Learning-induced LTP in neocortex. Science 290, 533–536. 10.1126/science.290.5491.533 11039938

[B39] Rioult-Pedotti M. S. Friedman D. Hess G. Donoghue J. P. (1998). Strengthening of horizontal cortical connections following skill learning. Nat. Neurosci. 1, 230–234. 10.1038/678 10195148

[B40] Rosenkranz K. Kacar A. Rothwell J. C. (2007). Differential modulation of motor cortical plasticity and excitability in early and late phases of human motor learning. J. Neurosci. 27, 12058–12066. 10.1523/jneurosci.2663-07.2007 17978047 PMC6673358

[B41] Sanes J. N. Donoghue J. P. (2000). Plasticity and primary motor cortex. Annu. Rev. Neurosci. 23, 393–415. 10.1146/annurev.neuro.23.1.393 10845069

[B42] Schnitzler A. Salenius S. Salmelin R. Jousmäki V. Hari R. (1997). Involvement of primary motor cortex in motor imagery: a neuromagnetic study. Neuroimage 6, 201–208. 10.1006/nimg.1997.0286 9344824

[B43] Shin H. W. Kang S. Y. Hallett M. Sohn Y. H. (2012). Reduced surround inhibition in musicians. Exp. Brain Res. 219, 403–408. 10.1007/s00221-012-3102-z 22543743 PMC4161923

[B44] Solodkin A. Hlustik P. Chen E. E. Small S. L. (2004). Fine modulation in network activation during motor execution and motor imagery. Cereb. Cortex 14, 1246–1255. 10.1093/cercor/bhh086 15166100

[B45] Stefan K. Kunesch E. Benecke R. Cohen L. G. Classen J. (2002). Mechanisms of enhancement of human motor cortex excitability induced by interventional paired associative stimulation. J. Physiol. 543, 699–708. 10.1113/jphysiol.2002.023317 12205201 PMC2290505

[B46] Stefan K. Kunesch E. Cohen L. G. Benecke R. Classen J. (2000). Induction of plasticity in the human motor cortex by paired associative stimulation. Brain 123(Pt. 3), 572–584. 10.1093/brain/123.3.572 10686179

[B47] Stefan K. Wycislo M. Gentner R. Schramm A. Naumann M. Reiners K.. (2006). Temporary occlusion of associative motor cortical plasticity by prior dynamic motor training. Cereb. Cortex 16, 376–385. 10.1093/cercor/bhi116 15930370

[B48] Stinear C. M. Byblow W. D. Steyvers M. Levin O. Swinnen S. P. (2006). Kinesthetic, but not visual, motor imagery modulates corticomotor excitability. Exp. Brain Res. 168, 157–164. 10.1007/s00221-005-0078-y 16078024

[B49] Toyoizumi T. Pfister J.-P. Aihara K. Gerstner W. (2005). Generalized Bienenstock-Cooper-Munro rule for spiking neurons that maximizes information transmission. Proc. Natl. Acad. Sci. U S A 102, 5239–5244. 10.1073/pnas.0500495102 15795376 PMC555686

[B50] Vahdat S. Darainy M. Ostry D. J. (2014). Structure of plasticity in human sensory and motor networks due to perceptual learning. J. Neurosci. 34, 2451–2463. 10.1523/JNEUROSCI.4291-13.2014 24523536 PMC3921420

[B51] Vargas C. D. Olivier E. Craighero L. Fadiga L. Duhamel J. R. Sirigu A. (2004). The influence of hand posture on corticospinal excitability during motor imagery: a transcranial magnetic stimulation study. Cereb. Cortex 14, 1200–1206. 10.1093/cercor/bhh080 15142965

[B52] Williams J. Pearce A. J. Loporto M. Morris T. Holmes P. S. (2012). The relationship between corticospinal excitability during motor imagery and motor imagery ability. Behav. Brain Res. 226, 369–375. 10.1016/j.bbr.2011.09.014 21939692

[B53] Willingham D. B. (1998). A neuropsychological theory of motor skill learning. Psychol. Rev. 105, 558–584. 10.1037//0033-295x.105.3.558 9697430

[B54] Wolpert D. M. Flanagan J. R. (2001). Motor prediction. Curr. Biol. 11, R729–R732. 10.1016/S0960-9822(01)00432-8 11566114

[B55] Wolpert D. M. Miall R. C. Kawato M. (1998). Internal models in the cerebellum. Trends Cogn. Sci. 2, 338–347. 10.1016/S1364-6613(98)01221-2 21227230

[B56] Wolters A. Sandbrink F. Schlottmann A. Kunesch E. Stefan K. Cohen L. G.. (2003). A temporally asymmetric Hebbian rule governing plasticity in the human motor cortex. J. Neurophysiol. 89, 2339–2345. 10.1152/jn.00900.2002 12612033

[B59] World Medical Association (1964). Declaration of Helsinki.

[B57] Zhang H. Long Z. Ge R. Xu L. Jin Z. Yao L.. (2014). Motor imagery learning modulates functional connectivity of multiple brain systems in resting state. PLoS One 9:e85489. 10.1371/journal.pone.0085489 24465577 PMC3894973

[B58] Ziemann U. Ilić T. V. Pauli C. Meintzschel F. Ruge D. (2004). Learning modifies subsequent induction of long-term potentiation-like and long-term depression-like plasticity in human motor cortex. J. Neurosci. 24, 1666–1672. 10.1523/jneurosci.5016-03.2004 14973238 PMC6730462

